# The Eukaryote-Like Serine/Threonine Kinase STK Regulates the Growth and Metabolism of Zoonotic *Streptococcus suis*

**DOI:** 10.3389/fcimb.2017.00066

**Published:** 2017-03-07

**Authors:** Chunyan Zhang, Wen Sun, Meifang Tan, Mengmeng Dong, Wanquan Liu, Ting Gao, Lu Li, Zhuofei Xu, Rui Zhou

**Affiliations:** ^1^State Key Laboratory of Agricultural Microbiology, College of Veterinary Medicine, Huazhong Agricultural UniversityWuhan, China; ^2^Veterinary Medicine Laboratory, Institute of Animal Husbandry and Veterinary Medicine, Jiangxi Academy of Agricultural SciencesNanchang, China; ^3^Veterinary Medicine Laboratory, Institute of Animal Husbandry and Veterinary Science, Hubei Academy of Agricultural SciencesWuhan, China; ^4^Cooperative Innovation Center of Sustainable Pig ProductionWuhan, China

**Keywords:** *Streptococcus suis*, eukaryote-like serine/threonine kinase, phosphorylation, RNA-Seq, phosphoproteome, growth, virulence, metabolism

## Abstract

Like eukaryotes, bacteria express one or more serine/threonine kinases (STKs) that initiate diverse signaling networks. The STK from *Streptococcus suis* is encoded by a single-copy *stk* gene, which is crucial in stress response and virulence. To further understand the regulatory mechanism of STK in *S. suis*, a *stk* deletion strain (Δ*stk*) and its complementary strain (CΔ*stk*) were constructed to systematically decode STK characteristics by applying whole transcriptome RNA sequencing (RNA-Seq) and phosphoproteomic analysis. Numerous genes were differentially expressed in Δ*stk* compared with the wild-type parental strain SC-19, including 320 up-regulated and 219 down-regulated genes. Particularly, 32 virulence-associated genes (VAGs) were significantly down-regulated in Δ*stk*. Seven metabolic pathways relevant to bacterial central metabolism and translation are significantly repressed in Δ*stk*. Phosphoproteomic analysis further identified 12 phosphoproteins that exhibit differential phosphorylation in Δ*stk*. These proteins are associated with cell growth and division, glycolysis, and translation. Consistently, phenotypic assays confirmed that the Δ*stk* strain displayed deficient growth and attenuated pathogenicity. Thus, STK is a central regulator that plays an important role in cell growth and division, as well as *S. suis* metabolism.

## Introduction

Upon sensing external stimuli, protein kinases, together with their cognate phosphatases, play a central role in signal transduction to quickly respond and adapt to constantly changing environments in both prokaryotes and eukaryotes. Reversible phosphorylation occurs on specific amino acid residues, most commonly serine (Ser), threonine (Thr), tyrosine (Tyr), histidine (His), and aspartate (Asp) (Pereira et al., 2011). Earlier classifications of prokaryote, kinases have been assumed to target only residues His and Asp, which are involved in two-component systems (TCS; Stock et al., 1990; Hoch, 2000). Increasing attention has been paid to the Ser/Thr kinases and their partner phosphatases. Some bacterial Ser/Thr kinases, which show conservation in their catalytic domains compared with eukaryotic Ser/Thr kinases, are designated as “eukaryote-like Ser/Thr kinases (eSTK)” (Pereira et al., 2011). Pkn1 of *Myxococcus xanthus* is the first characterized eSTK in bacteria (Muñoz-Dorado et al., 1991); subsequently a second eSTK, Pkn2, has been identified in *M. xanthus* (Udo et al., 1995). Furthermore, numerous bacterial eSTKs have been identified based on genome sequence databases (Galperin et al., 2010). Multiple eSTKs exist in most bacteria; therefore, comprehensively characterizing their essentiality and identifying their specific substrates are difficult. For example, 11 eSTKs exist in *Mycobacterium tuberculosis* that has functional redundancy and/or substrate promiscuity (Boitel et al., 2003; Sajid et al., 2015).

The eSTKs have been widely studied for their roles in diverse biological processes, including development (Zhang, 1993; Nádvorník et al., 1999; Inouye and Nariya, 2008), cell competence (Hussain et al., 2006), cell division, and cell wall synthesis (Deol et al., 2005; Fernandez et al., 2006; Ruggiero et al., 2012), central and secondary metabolism (Lee et al., 2002; Sawai et al., 2004), biofilm formation (Hussain et al., 2006; Liu et al., 2011), stress response (Neu et al., 2002; Mata-Cabana et al., 2012), and virulence (Madec et al., 2002; Rajagopal et al., 2003; Echenique et al., 2004). Gene expression profiles have proven their global regulatory roles in cellular processes (Sasková et al., 2007; Donat et al., 2009). Moreover, both phosphoproteomic analyses and kinase assays have identified eSTK substrates in *Streptococcus pyogenes* (Jin and Pancholi, 2006), *Streptococcus pneumoniae* (Nováková et al., 2005, 2010), *Streptococcus agalactiae* (Silvestroni et al., 2009), *Staphylococcus aureus* (Lomas-Lopez et al., 2007; Truong-Bolduc et al., 2008), *Listeria monocytogenes* (Archambaud et al., 2005), and *M. tuberculosis* (Arora et al., 2010). Most identified substrates are involved in cell growth/division and central metabolism of bacteria. Various microorganisms have been studied, but the profound effects of eSTKs and posttranslational modification on their targets remain poorly understood.

*Streptococcus suis* is a zoonotic Gram-positive pathogen that causes lethal infections in pigs and humans (Lun et al., 2007). Two large outbreaks of human *S. suis* infection have been reported in 1998 and 2005 in China, resulting in 229 infections and 52 deaths (Lun et al., 2007). Among the 33 *S. suis* serotypes, serotype 2 (SS2) is the most virulent and prevalent serotype isolated from diseased pigs (Smith et al., 1999). Moreover, SS2 is the prominent agent that caused adult human meningitis in Vietnam and Hong Kong (Wertheim et al., 2009). Numerous virulence-associated factors of *S. suis* have been identified over the past decade, such as capsular polysaccharide, muramidase-released protein, suilysin, extracellular factor, fibrinonectin- and fibrinogen-binding proteins, enolase, arginine deiminase system, glyceraldehyde-3-phosphate dehydrogenase (GAPDH), inosine 5-monophosphate dehydrogenase (IMPDH), secreted nuclease A (SsnA), subtilisin-like protease A (Fittipaldi et al., 2012), H binding protein (Fhb; Pian et al., 2012), and so on. Compared with other Gram-positive bacteria, only a single-copy *stk* is present in the *S. suis* genome (Zhu et al., 2014). The STK of *S. suis* is involved in stress response and virulence. The disruption of *stk* in *S. suis* enables increased chain-length, reduced tolerance to high temperature, low acidic pH, oxidative stress, and decreased virulence (Zhu et al., 2014).

To further understand the regulatory mechanism of STK in *S. suis*, we constructed a *stk*-deletion mutant (Δ*stk*) and investigated its biological characterizations using “-omics” approaches. By comparing the transcriptomic profiles, we identified differentially expressed genes (DEGs) between the Δ*stk* strain and the wild-type parental strain, SC-19. Using phosphoproteome analyses, phosphorylation level of protein-coding sequences were systematically estimated. The analyses of both transcriptomic and phosphoproteomic provide functional context that STK can regulate cell growth and division, as well as metabolism of *S. suis*.

## Materials and methods

### Bacterial strains, plasmids, and culture conditions

The bacterial strains and plasmids used in this study are listed in Table 1. The virulent *S. suis* strain SC-19 was isolated from a diseased pig during the 2005 outbreak in Sichuan, China (Li et al., 2009). Since the genome of SC-19 has not been sequenced, the genome sequence of the strain *S. suis* 05ZYH33 (GenBank accession number CP000407) was used as reference for gene clone, transcriptomic, and phosphoproteomic analysis. *S. suis* 05ZYH33 was isolated from an infected human during the same outbreak in Sichuan (Lun et al., 2007). Both of these two isolates are serotype 2. Bacteria were grown in TODD-Hewitt broth (THB; OXOID, England) medium or plated on THB Agar (THA; OXOID) with 5% (v/v) sheep blood at 37°C. Erythromycin (90 μg/ml) was added to screen the mutant strain and erythromycin (90 μg/ml) and spectinomycin (100 μg/ml) were added to select for a complementary strain.

**Table 1:** **Bacterial strains and plasmids used in this study**.

**Stains and plasmids**	Revelant characteristics and genotype^*^	**Sources or references**
***S. suis* STRAINS**		
SC-19	*S. suis* serotype 2, the wild-type (Strep^r^)	Li et al., 2009
Δ*stk*	SC-19 *stk*::erm (Strep^r^ Erm^r^)	This study
CΔ*stk*	SC-19 *stk*::erm/*stk*^+^ (Strep^r^ Erm^r^ Spc^r^)	This study
***E. coli***		
DH5α	Cloning host for recombinant vector	Trans
BL21 (DE3)	Expressing host for fusion protein	Trans
**PLASMIDS**		
pET-28a	Expression vector (Kan^r^)	Novagen
pSTK	pET-28a containing *stk*, cloned from SC-19 genome	This study
pAT18	A plasmid carrying an erythromycin resistance rRNA methylase (*erm*) gene expression cassette	Trieu-Cuot et al., 1991
pSET4s	Temperature-sensitive *E. coli*-*S. suis* shuttle vector (Spc^r^)	Takamatsu et al., 2001b
pSET4s-S	Derived from pSET4s for deleting *stk* in SC-19	This study
pSET2	*E. coli*-*S. suis* shuttle vector (Spc^r^)	Takamatsu et al., 2001a
pSET2-CM	Derived from pSET2 for functional complementation of *stk* (Spc^r^)	This study

* *Strep^r^, streptomycin resistant; Erm^r^, erythromycin resistant; Spc^r^, spectinomycin resistant; Kan^r^, kanamycin resistant*.

*Escherichia coli* (*E. coli*) strain DH5α (Trans, China) was used as host strain for cloning and *E. coli* strain BL21 (DE3) (Trans) was used to express His-tag fusion proteins. *E. coli* strains were cultured in Luria-Bertani (LB) Broth (Difco, France) or plated on LB Agar at 37°C. If necessary, kanamycin (25 μg/ml) was added.

### Construction of mutant and complementary strain

The *stk* deletion strain was obtained using an existing method (Takamatsu et al., 2001b; Zhu et al., 2014). Primers used in this study were designed according to the genome sequence of *S. suis* 05ZYH33 and are listed in Table S1. Primers SU-F/SU-R and SD-F/SD-R were used to amplify the upstream and downstream regions of *stk*. Moreover, the fragments were cloned into pSET4s, respectively. Finally, the erythromycin-resistance expression cassette (*erm*^R^) was amplified from pAT18 with primers Erm-F/Erm-R and cloned to achieve the *stk*-knockout vector pSET4s-S. The pSET4s-S was then electroporated into SC-19. Through homologous recombination, the *stk* gene was replaced the *erm*^R^ via double-crossover incident.

The promoter sequence of IMPDH was selected to drive the expression of *stk* gene in SC-19 (Takamatsu et al., 2001a; Zhu et al., 2014). The promoter and the coding sequence of *stk* were cloned into pSET2 to obtain the recombinant plasmid pSET2-S. The pSET2-S was transformed into the mutant strain Δ*stk* to acquire the complementary strain CΔ*stk*.

To further confirm the mutant strain Δ*stk* and the complementary strain CΔ*stk*, we performed Western blot analysis. The overnight cultures of SC-19, Δ*stk* and CΔ*stk*, cultured in THB were collected by centrifugation and resuspended in bacterial lysis buffer (50 mM Tris/HCl, pH 8.5, 100 mM NaCl, 2 mM ethylene diamine tetraacetic acid, 100 μg/ml lysozyme, 1 mM phenylmethanesulfonyl fluoride, 0.5% Triton X-100). Bacterial cells were lysed with a French pressure cell press. Quantified bacterial lysate was separated on SDS-PAGE and then electrotransferred to PVDF membrane (Invitrogen, USA) (Tan et al., 2015). Mouse anti-STK serum was produced as described previously (Li et al., 2011) by using recombinant STK protein. The PVDF membranes (Invitrogen, USA) were probed with primary antibodies against STK (1:1000) or GidA as control (1:1000; stored at −80°C in our laboratory). After wash, the membranes were incubated with goat anti-mouse IgG (H+L)-HPR (1:5000; Southern Biotech, USA). Antibody-tagged protein bands were detected by using Western ECL Substrate Kit (Bio-Rad, USA).

### RNA extraction and RT-PCR

To confirm the mutant strain Δ*stk* and the complementary strain CΔ*stk*, we also performed RT-PCR (Tan et al., 2015). Briefly, RNA was isolated using SV Total RNA Isolation System (Promega, USA) according to the manufacturer's instructions. Then, cDNA was synthesized using HiScript Q Select RT SuperMix (Vazyme, China) according to the manufacturer's instructions.

To confirm whether the upstream and downstream genes of *stk* in the mutant were affected or not, the genes were amplified using the primers SSU05_0427-F/SSU05_0427-R (for upstream gene) and SSU05_0429-F/SSU05_0429-R (for downstream gene; Table S1) using the SC-19 cDNA as template.

### Protein expression and purification

The coding sequence of *stk* was amplified using primers *stk*-F/*stk*-R from the SC-19 genome. The PCR product was restricted with EcoRI and XhoI and then inserted into the digested pET-28a vector to generate the recombinant plasmid pET-*stk*, which was then transformed into *E. coli* BL21 (DE3) cells. Expression was induced by 1 mM isopropyl-β-D-thiogalactopyranoside (IPTG) (Sigma, USA) at 18°C for 12 h. His-tagged STK was purified using Ni-NTA columns (GE Healthcare, Sweden) according to the manufacturer's recommendation. The purified protein was identified by Western blot analysis using the His-tag rabbit-polyclonal antibody (Sigma, USA) and STK-mouse polyclonal antibody (made in our laboratory), respectively.

### Protein kinase assay

The kinase assay was performed as described previously (Boitel et al., 2003; Fernandez et al., 2006). The reaction was conducted in 50 μl of kinase buffer (50 mM Hepes, 1 mM DTT, 0.01% Brij35, pH 7.0) containing 2 mM MnCl_2_, 100 μM ATP and 1 μCi of [γ-^32^P]-ATP (PerkinElmer, UK). The reaction was initiated by adding the kinase at 30°C for 10 min and stopped by adding SDS-PAGE sample buffer plus EDTA (25 mM final). A total of 20 μl of the reaction was subjected to electrophoresis. In each case, the reaction products were separated on a 12% SDS-polyacrylamide gel and the radiolabelled proteins were visualized using auto-radiography. To obtain relative quantification of the radiolabelled ATP incorporation, the radioactive samples were analyzed using a PhosphorImager apparatus (Fujifilm, Japan). For the substrate phosphorylation, myelin basic protein (MBP; Sigma, USA) was used as a positive control to test the kinase activity. The enzyme/substrate ratio was 1:10 with 0.4 μM kinase.

### Microscopy image

The strains SC-19, Δ*stk* and CΔ*stk* at the mid-log phase were collected and then resuspended twice using ddH_2_O. Each sample (20 μl) was fixed on glass slides (Shitai, China) through flaming. A Gram staining kit (Jiancheng, China) was utilized according to the manufacturer's instruction. The stained samples were observed under light microscope.

Scanning electron microscope (SEM) observation was performed following previous methods (Wang et al., 2011; Fleurie et al., 2012). The bacteria were grown in THB broth at 37°C and harvested at OD_600_ of 0.6. Bacterial suspensions were spotted onto glass coverslips (0.17 mm in thickness and 20 mm in diameter; WHB, China) and washed with phosphate-buffered saline. The cells were fixed with 2.5% glutaraldehyde overnight at 4°C. All samples were dehydrated with a series of gradient ethanol and air dried. The dried samples were covered with a 10 nm gold/platinum layer (JSM-6390LV, JEOL, Japan) and observed via SEM (JFC-1600, JEOL, Japan).

All SEM images were analyzed with Image J. The sizes (in pixels) of bacterial chains were defined as their two-dimensional area based on the “Auto Threshold” function, which defined the cell chain outline. Shapes that were not chains and chains on the border of images were manually excluded from subsequent analyses (Dalia and Weiser, 2011). For chain length analysis, data are the result of at least 100 chains analyzed per sample from at least 100 fields.

### Detection of growth characterizations

Growth rates of the SC-19, Δ*stk* and CΔ*stk* were detected through the measurement of the density changes represented by OD_600_ nm values and CFU counts of the cultures. Different strains were grown overnight in THB medium and then the initial OD_600_ nm of all subcultures were adjusted to 0.014. The diluted cells were incubated at 180 rpm/m at 37°C in the same medium and OD_600_ nm values were read every hour until the growth process entered stationary phase. Meanwhile, 100 μl of the bacterial culture was diluted and then vortexed to break the chains. Bacteria numbers were recorded every hour by viable count. All the growth data were analyzed with Origin 8.0. The growth rate at each time point was calculated based on the function of “Non-linear Curve Fit” and “Mathematics.” The average growth rate (AGR) was calculated from data in mid-log phase based on the function of “Linear Curve Fit” (OriginLab, Northampton, MA, USA).

### Virulence assay in mice

All mice used in our study were purchased from the Wuhan Institute of Biological Products (Wuhan, China). The study was performed strictly in accordance with the Ethics Committee of Huazhong Agricultural University according to Hubei Province Laboratory Animal Management Regulations-2005. All efforts were made to minimize suffering.

To probe the possible role of the *stk* gene in *S. suis* virulence, 24 female specific-pathogen-free (SPF) Kunming mice (4–6 weeks old) were divided into three groups. Groups 1 and Group 2 were intraperitoneally injected with 1 × 10^9^ colony-forming unit (CFU) mid-log-phase cells of either SC-19 or Δ*stk*. Saline was applied in Group 3 as negative control. Clinical signs and survival time were recorded. The mice were observed for 7 days to obtain steady survival curves.

To better evaluate the pathogenicity of Δ*stk*, we performed a determination of viable bacteria in organs assay as described previously (Gao et al., 2016). Six-week-old female SPF Kunming mice (6 mice per group) were intraperitoneally inoculated with 5 × 10^8^ CFU of a 1:1 mixture of mid-log phase SC-19 and Δ*stk*. Saline was applied as negative control in six mice. At 6, 18, and 52 h post-infection (hpi), blood samples, brains, and lungs were obtained from each group. The samples were homogenized after weighing, and serial dilutions were plated onto THA. To count the colonies, we used streptomycin (20 μg/ml) for SC-19, whereas 20 μg/ml streptomycin and 90 μg/ml erythromycin were used for Δ*stk*.

### RNA-Seq analysis

To investigate gene expression profiles between SC-19 and Δ*stk*, RNA-Seq was performed in the BGI, Shenzhen. Three biological replicates were mixed as one sample before RNA-Seq. RNA isolation and purification were the same as aforementioned. RNA-Seq based transcriptomic profiles were conducted as previous cited (Wilhelm and Landry, 2009). After the quality inspection using an Agilent 2100 Bioanalyzer and ABI StepOnePlus Real-Time PCR System, the library was sequenced using Illumina HiSeq 2000. Quality control of raw reads was performed and clean reads were mapped onto the complete reference *S. suis* 05ZYH33 genome.

The gene expression level was calculated by using RPKM (reads per kb per million reads) method. The number of reads that uniquely aligned to a unique gene was normalized to RPKM (Mortazavi et al., 2008). The RPKM method eliminates the effect of different gene lengths and sequencing levels on the calculation of gene expression. Therefore, the gene expression can be directly calculated by comparing the different gene expression level among samples (Chen et al., 2014). To identify DEGs between two samples, a statistical analysis of the frequency of each unique-match read in each library was performed by referring to “the significance of digital gene expression profiles” (Audic and Claverie, 1997; Chen et al., 2014). The probability of gene A expressed equally between two samples can be calculated with the following formula, wherein the total clean tag number of the sample 1 is N_1_, and total clean tag number of sample 2 is N_2_; gene A holds *x* tags in sample 1 and *y* tags in sample 2; *p*-value corresponds to differential gene expression test (Audic and Claverie, 1997; Chen et al., 2014).

$$\begin{array}{l} 2\overset{y}{\underset{i=0}{\sum }}p\left(i/x\right)\left(while\;\overset{y}{\underset{i=0}{\sum }}p\left(i/x\right)\;\leq 0.5\right)\; \end{array}$$

or

$$\begin{array}{l} 2\times \left(1-\overset{\text{y}}{\underset{\text{i}=0}{\sum }}p\left(i/x\right)\right)\;(while\;\overset{\text{y}}{\underset{\text{i}=0}{\sum }}p\left(i/x\right)>0.5)\\ p\left(i/x\right)={\left(\frac{{\text{N}}_{2}}{{\text{N}}_{1}}\right)}^{i}\frac{\left(x+i\right)!}{x\;!\;i!\;{\left(1+\frac{\text{N}2}{\text{N}1}\right)}^{\left(x+i+1\right)}} \end{array}$$

False Discovery Rate (FDR) was used in the multiple hypothesis testing to correct for *p*-value (Chen et al., 2014). Following the formula below, assuming W DEGs had been selected, M genes of those were really differential expressed, whereas H genes indicated no difference which were false positive. If we decide that the error ratio “Q = H/W” must stay below a cutoff (e.g., 5%), we should preset the FDR to a number no larger than 0.05. FDR-values were calculated according to the previous algorithm (Li J. et al., 2015)

$$\begin{array}{l} \text{FDR}=\text{E}\left(\text{Q}\right)=\text{E}\left\{\text{H}/\left(\text{H}+\text{M}\right)\right\}=\text{E}\left(\text{H}/\text{W}\right) \end{array}$$

To detecte DEGs, we used FDR ≤ 0.001 and log transformed fold change (the ratio of RPKM values) > 2.0. Moreover, enrichment analysis based on KEGG pathway database (http://www.genome.jp/kegg/) was done using the R package GAGE v2.22 (Luo et al., 2009). Pathway-level differential expression was visually checked using Pathview (Luo and Brouwer, 2013). Functional classification of gene products was predicted using BLASTP v2.5.0+ (Altschul et al., 1997) by the clusters within the orthologous group (COG) database v2014 (Galperin et al., 2015). The *q*-value cutoff for BLAST searching was set to 0.1. Putative virulence-associated genes (VAGs) of *S. suis* were screened by searching all protein sequences against Virulence Factor Database (VFDB; Chen et al., 2005) using BLASTP. The *E*-value cutoff for BLAST searching was set to 1e-20. The RNA-Seq data have been submitted to the NCBI Gene Expression Omnibus (GEO) database under the accession number GSE87759.

### Quantitative RT-PCR (qRT-PCR)

A subgroup (*n* = 10) of DEGs was selected to cross-validate the RNA-Seq data with SYBR green detection. The primers (Table S1) were designed according to the genomic sequence of *S. suis* 05ZYH33. RNA extraction was carried out as described above. qRT-PCR was performed on an ABI 7300 HT Sequence Detection System using the ABI Power SYBE Green PCR Master Mix. The 16S rRNA served as an internal reference gene. The relative expression level was measured using the 2^−ΔΔ*ct*^ method. Data were reported as mean relative expression levels (± standard deviation) between SC-19 and Δ*stk*. Five depressed genes in Δ*stk* (SSU05_1776, SSU05_0272, SSU05_1815, SSU05_0309, and SSU05_2154) and five stimulative genes in Δ*stk* (SSU05_0792, SSU05_0358, SSU05_1011, SSU05_0906, and SSU05_1532) were chosen to do qRT-PCR for RNA-Seq confirmation. The assays were replicated thrice.

Subsequently, we performed qRT-PCR to compare the transtriptional levels of the above genes between SC-19 and CΔ*stk* related to Δ*stk*. The relative expression level was measured using the 2^−ΔΔ*ct*^ method. Data were reported as mean relative expression levels (± standard deviation) between SC-19 and Δ*stk*, as well as between CΔ*stk* and Δ*stk*.

### Protein extraction, digestion, labeling with iTRAQ reagents, and phosphopeptide enrichment

SC-19 and Δ*stk* cells at mid-log phase were cultured in THB as described above. Three independent biological replicates of bacterial pellets were then treated with SDT buffer (4% SDS, 100 mM Tris-HCl, 1 mM DTT, pH 7.6) and heated for 15 min at 100°C. The cell suspensions were sonicated for 5 min (10 s sonication with 15 s interval, 80 W) on ice and protein concentrations in the supernatants were determined through Bradford protein assay (Gao et al., 2016). Each sample (20 μg) was then prefractionated using 12% SDS-PAGE. Subsequently, the gel was processed using 200 μl UA buffer (8 M Urea, 150 mM Tris-HCl, pH 8.0) and subjected to in-gel tryspin digestion at 37°C for 16 h (Hu et al., 2014). The resulting tryptic peptides were labeled according to the protocol of iTRAQ Reagent-8 plex Multiplex Kit (Applied Biosystems, USA). The labeled peptides were mixed, concentrated using a vacuum concentrator and resuspended in 500 μl 1 × DHB buffer (3% DHB, 80% CAN, 0.1% TFA). Afterward, TiO_2_ beads were added and agitated for 40 min. The centrifugation was performed, resulting in the first beads. The supernatant from the first centrifugation was mixed with additional TiO_2_ beads, resulting in the second beads that were collected as before. Both bead groups were combined and washed thrice with 50 μl of washing buffer I (30% ACN, 3% TFA) and then washed thrice with 50 μl washing buffer II (80% ACN, 3% TFA) to remove the non-absorbed material. Finally, the phosphopeptides were eluted with 50 μl of elution buffer (40% ACN, 15% NH_4_OH), followed by lyophilization and MS analysis.

### LC-MC/MS analysis

Phosphopeptide solution (6 μl) was mixed with 0.1% (v/v) fluoroacetic acid (20 μl). Equal amounts of digested protein were loaded into a Thermo Scientific EASY column (2 cm^*^100 μm 5 μm-C18) and then washed with solvent A (99% H_2_O, and 0.1% formic acid). By applying solvent B (84% acetonitrile, 16% H_2_O, and 0.1% formic acid), the peptides were eluted from the trapping column over a Thermo scientific EASY column (75 μm* 100 mm 3 μm-C18) with a gradient (0–55% B for 220 min at 250 nL/min, 55–100% B for 8 min, and 100% B for 12 min) using Thermo scientific Easy nLC system. For MS analysis, peptides were analyzed in positive ion mode. MS/MS was carried out with a Q-Exactive mass spectrometer (Thermo Finnigan, USA) setting in a positive ion mode and data-dependent manner choosing the most abundant precursor ions using a full MS scan from *m/z* 350–1800, with a resolution of 70,000 at *m/z* 200. Target value determination was based on automatic gain control and dynamic exclusion duration was 30 s. MS/MS scan was acquired at a resolution of 17,500 at *m/z* 200. Normalized collision energy was 29 eV and the underfill ratio was set to 0.1%. Quantitation was achieved by comparing the peak areas and resultant peak ratios among the four MS/MS reporter ions from 114 to 117 Da.

### Phosphoproteomic data analysis

The data files produced by LC-MS/MS were processed by Proteome Discoverer 1.4 and searched by Mascot 2.2 (Matrix Science, MA) against 26,496 *S. suis* protein-coding sequences deposited in the Uniprot database (downloaded on August 4, 2015). For peptides after phosphopeptide enrichment, the following options were used. Peptide mass tolerance: ± 20 ppm; MS/MS tolerance: 0.1 Da; enzyme: trypsin; max missed cleavages: 2; fixed modifications: carbamidomethyl (C), iTRAQ8plex (K) and iTRAQ8plex (N-term); variable modifications: oxidation (M), phosphorylation (Ser/Thr/Tyr). For peptides, proteins, and phosphosites identification, FDR was estimated and the threshold was set to 1%. The peptides were determined as true phosphorylation based on the following criteria: Phospho RS score > 50 and Phospho RS site probabilities > 75%. Proteome Discoverer 1.4 software was used to extract the peak intensity within 20 ppm of each expected iTRAQ reporter ion from each analyzed fragmentation spectrum. Only spectra in which all the expected iTRAQ reporter ions detected were used for quantification.

The intensity of the reporter ions was used for phosphopeptide quantification. We normalized the phosphopeptide ratios by dividing by the median ratio of all peptides identified. As for the quantitative analysis, the log_2_ fold-change values (SC-19/Δ*stk*) were calculated for each phosphopeptide. Only phosphopeptides detected in at least two out of the three biological replicates were used for assessment of significant change. The *t*-test was employed to identify significant changes between the wild type strain and the mutant strain among the three biological replicates. The phosphopeptides that passed *t*-test with *p* < 0.05 were considered to be significantly regulated (Fan et al., 2014). We also included the cutoff for the log_2_ fold change values, in which the phosphorylation changes were considered highly significant if the log_2_ value ≥ 0.26 or ≤ −0.26. The data were then normalized and logarithmically transformed as FC. FC (Δ*stk*/SC-19) ≥ 1.2 and ≤ 0.83 were used to represent up- or down-regulations. The mass spectrometry proteomics data have been deposited to the ProteomeXchange Consortium via the PRIDE (Vizcaino et al., 2016) partner repository with the dataset identifier PXD005663.

### Statistical analysis

The means of two groups were compared using Student's *t*-test (unpaired, two-tailed) in GraphPad Prism 5 (San Diego, USA), with *p* < 0.05 considered to be statistically significant. Unless otherwise specified, all the experiments were performed in triplicate at least thrice.

## Results

### Characterization of STK in *S. suis*

**Figure 1:**
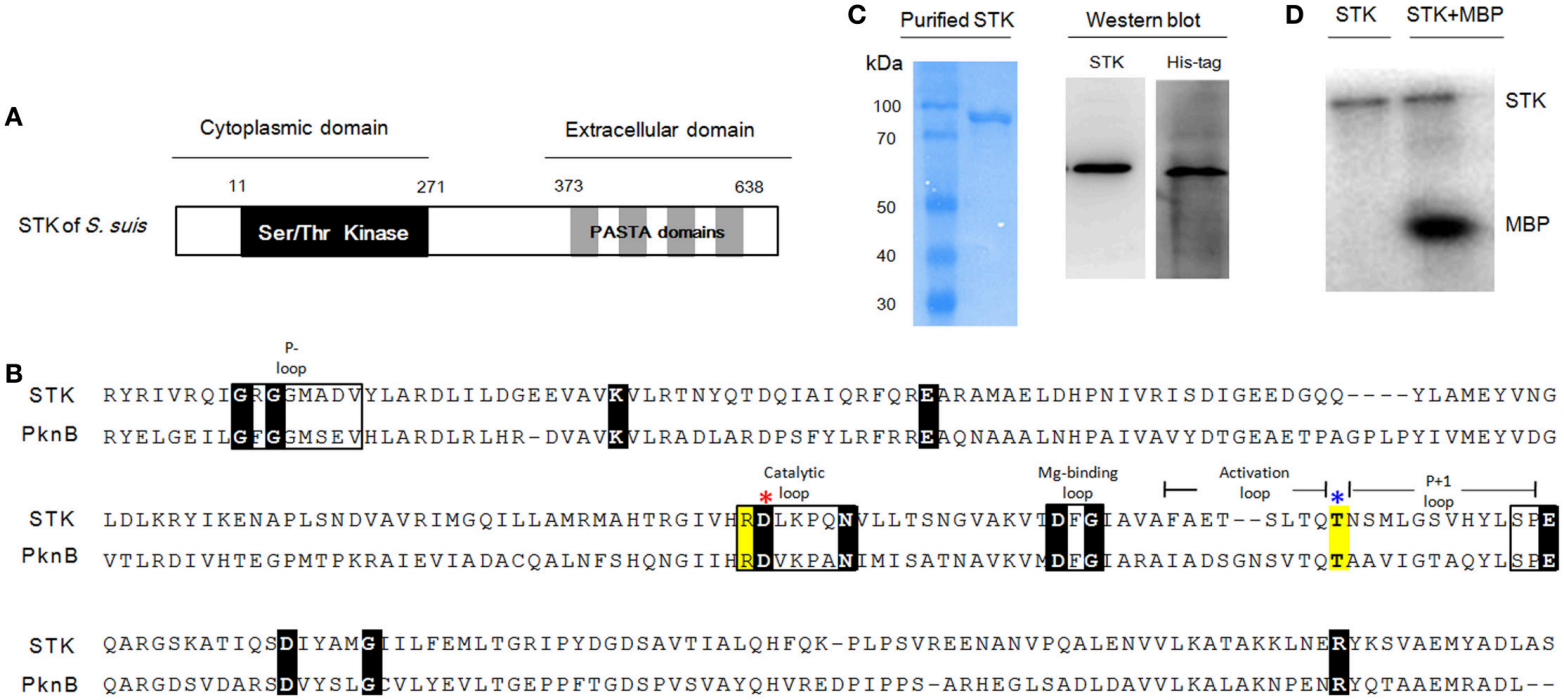
**Kinase domain of STK in *S. suis* is conserved compared with other genera. (A)** The predicted secondary structure of the STK protein. **(B)** Multiple sequence alignments of *S. suis* STK and *M. tuberculosis* PknB. Sequences used here are separately collected from *S. suis* 05ZYH33 (CP000407) and *M. tuberculosis* H37Rv (NC_000962.3). Conserved motifs are shown in boxes and the invariant residues are marked with black bottom color. Other important residues are highlighted in yellow. Red and blue asterisks indicate the catalytic Asp and phosphorylated Thr residues, respectively. **(C)** SDS-PAGE and Western blot analysis of STK. **(D)** Kinase activity of STK *in vitro*. Left lane, STK was incubated with [γ-^32^P]-ATP; Right lane, STK and MBP were mixed and incubated with [γ-^32^P]-ATP.

As previously described, the STK in *S. suis* consists of a cytoplasmic kinase domain (residues 11–267 aa) and an extracellular C-terminal region composed of four penicillin-binding proteins (residues 373–434 aa; 441–500 aa; 507–568 aa; 575–638 aa) and a Ser/Thr kinase-associated domain (PASTA; residues 348–629 aa; Figure 1A; Beilharz et al., 2012). Protein sequence alignments between STK and its homolog PknB (Rv0014c in *M. tuberculosis* H37Rv; NC_000962.3; Boitel et al., 2003) showed that Ser/Thr kinase domains share 43% similarity (Figure 1B). The STK expression was confirmed by Western blot analysis (Figure 1C). The *ex vivo* kinase assay demonstrated that STK possesses autophosphorylation and substrate phosphorylation activities (Figure 1D).

### Construction and characterization of Δ*stk* and CΔ*stk*

We constructed a deletion mutant of *stk*, as confirmed by RT-PCR (Figure S1A) and Western blot (Figure S1B). The RT-PCR showed that the *stk* upstream and downstream genes transcriptions were not affected by *stk* deletion. Western blot showed the STK protein did not expressed in Δ*stk*, but presented in SC-19 and CΔ*stk*.

**Figure 2:**
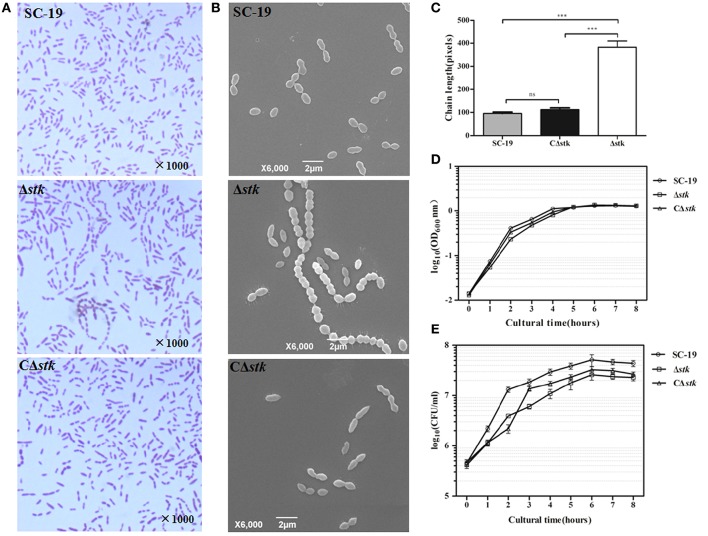
**Micrographs and growth curves of SC-19, Δ*stk*, and CΔ*stk*. (A)** Light microscope morphology of *S. suis* using Gram staining. **(B)** Scanning electron micrographs of the strains. **(C)** The average size of bacterial chains. Data are the result of at least 100 chains analyzed per sample ± Standard Error of Mean. The mutant strain Δ*stk* showed significantly increased chain sizes compared with those of SC-19 (^***^p < 0.001) and CΔ*stk* (^***^p < 0.001). **(D)** Growth curves of the strains. Bacterial cell density was measured spectrometrically at 600 nm. During the exponential phase (2–4 h), AGRs of the three strains displayed no significant difference (SC-19 was 0.51 % per min; AGR of Δ*stk* and CΔ*stk* was 0.49 % per min). **(E)** CFU count of the strains. The CFU counts showed that Δ*stk* grew much slower than SC-19 and CΔ*stk* during the exponential phase (2–4 h) (AGR of SC-19 was 1.47 × 10^5^ CFU per min; AGR of Δ*stk* was 6.58 × 10^4^ CFU per min; AGR of CΔ*stk* was 1.18 × 10^5^ CFU per min). Data are displayed on a logarithmic scale of Y-axis both in **(D)** and **(E)**. Results are expressed as log10 mean ± Standard Error of Mean OD or CFU/ml obtained from three independent experiments.

The effect of *stk* knockout on morphology of *S. suis* was investigated using Gram staining (Figure 2A) and SEM (Figure 2B). The results revealed that *stk* inactivation triggered significant chain elongation (Figure 2C).

To characterize SC-19, Δ*stk* and CΔ*stk*, we measured growth by detecting OD_600_ and CFU counts. The AGRs of mid-log phase (2–4 h) displayed no significant difference among the three strains detected by the OD_600_ value (AGR of SC-19 was 0.51% per min; AGR of Δ*stk* and CΔ*stk* was 0.49% per min; Figure 2D). Whereas, the AGRs of mid-log phase (2–4 h) detected by CFU counts showed that Δ*stk* grew much slower than SC-19 (AGR of SC-19 was 1.47 × 10^5^ CFU per min and Δ*stk* was 6.58 × 10^4^ CFU per min; Figure 2E). Although the growth ability of CΔ*stk* was not fully restored by introducing a complementary plasmid in the mutant, CΔ*stk* grew much faster than Δ*stk* during 2–4 h (AGR of CΔ*stk* was 1.18 × 10^5^ CFU per min).

We tried to use the endogenous promoter of *stk* gene to construct the complementary strain, but it failed (no expression; the endogenous promoter needs further characterization). Therefore, we used a known promoter in *S. suis* genome, the promoter of IMPDH (Zhu et al., 2014). Maybe this promoter is not a strong promoter to restore all the phenotypes. Similar facts have also been reported in other studies (Ju et al., 2012).

### Δ*stk* pathogenicity was attenuated in mice

**Figure 3:**
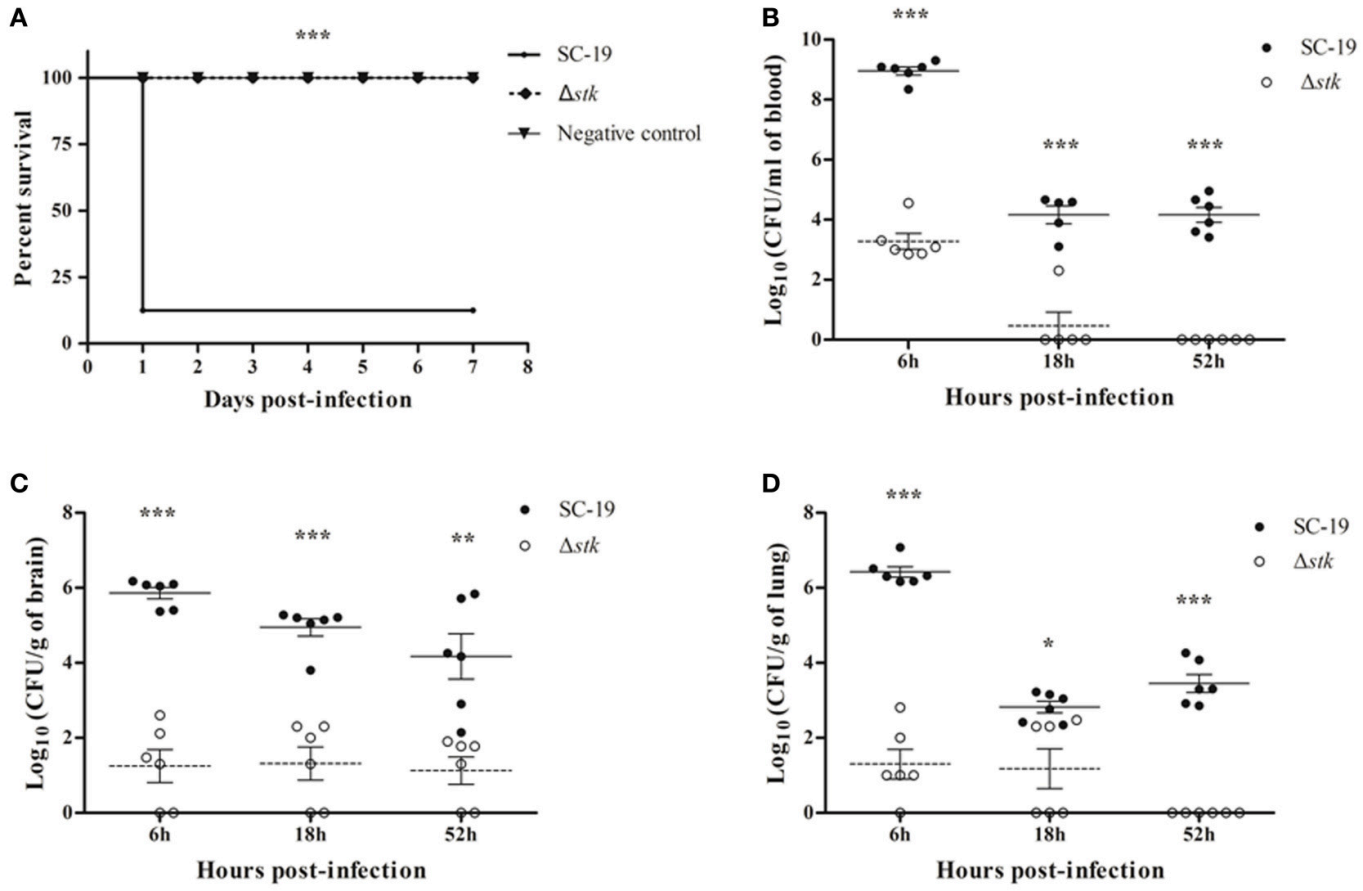
**Mouse infection models. (A)** Survival curves for mice in infection experiment. Eight mice in each group were separately injected intraperitoneally with 1 × 10^9^ CFU/mouse of SC-19 and Δ*stk*. Eight mice were inoculated with saline and served as negative control. Significant difference in survival between different groups was analyzed by Log Rank test (*p* < 0.001). **(B)** Bacteria loads in blood, **(C)** brain, and **(D)** lungs. Mice were inoculated intraperitoneally with 5 × 10^8^ CFU of a 1:1 mixture of mid-log phase SC-19 and Δ*stk*. The survival strains were enumerated by plating serial dilutions of the samples on selective plates. Data are the result of CFU/ml or CFU/g in different organs analyzed per sample ± Standard Error of Mean. Solid lines, the mean data of SC-19; dotted lines, the mean data of Δ*stk*. Statistical significance was determined using the two-tailed *t*-test (ns, *p* > 0.05; ^*^p < 0.05; ^**^p < 0.01; ^***^p < 0.001).

Experimental infection of mice was performed to estimate the difference of viability *in vivo* between Δ*stk* and SC-19. All SC-19-infected mice displayed severe clinical symptoms, such as septicemia, and most (7/8) died during the first day of infection. By contrast, all Δ*stk*-infected mice displayed slight clinical symptoms and had none mortality during the 7-day observation period (Figure 3A). Therefore, the pathogenicity of Δ*stk* was markedly attenuated.

To further evaluate the pathogenicity of Δ*stk*, we determined the differences of viable bacteria in organs using intraperitoneal inoculation. Bacteria were recovered from blood (Figure 3B), brains (Figure 3C), and lungs (Figure 3D) at different time points post-infection. The bacterial loads in blood, brains, and lungs were lower in Δ*stk* than SC-19, from 6 to 52 hpi. The mutant strain was almost cleared at the 52 hpi (Figures 3B–D). These results indicated that the survival ability of Δ*stk* in the host was weakened compared with the parental strain SC-19.

### DEGs of Δ*stk*

To get a glimpse of transcriptomic dysregulation of Δ*stk*, gene expression profiles were determined using RNA-Seq for Δ*stk* and SC-19. A total of 2,116 genes were expressed with read counts greater than five in at least one sample. Among these, 539 were differentially expressed (25% of expressed genes) in Δ*stk* compared with SC-19, with 320 up-regulated and 219 down-regulated (*p* < 0.05 and log transformed fold change > 2.0; Table S2). These genes were involved in diverse physiological activities, including carbohydrate metabolism, amino acid metabolism, nucleotide metabolism, and translation. As an internal control, *stk* transcript was not detected for Δ*stk* but presented in SC-19.

The effectiveness of the RNA-Seq data was cross-validated using qRT-PCR. Ten genes with various expression levels, as determined by RNA-Seq analysis were chosen for qRT-PCR analysis (Table 2). The correlation between the two methods was high (*R*^2^ = 0.903; Figure S2A).

**Table 2:** **Validation of RNA-Seq results by real-time quantitative RT-PCR (qRT-PCR)**.

**Gene locus_tag**	**Function**	**Fold change of RNA-Seq**	**Fold change of qRT-PCR**
SSU05_0272	Translation initiation factor 2	−3.47	−4.62 ± 1.00
SSU05_0309	Cation transport ATPase	−2.64	−3.48 ± 1.16
SSU05_0358	deoxyguanosinetriphosphate triphosphohydrolase-related protein	7.10	3.23 ± 1.23
SSU05_0792	Carbamoylphosphate synthase large subunit	17.58	14.17 ± 0.95
SSU05_0906	NisK	4.13	2.98 ± 1.01
SSU05_1011	dihydroorotate dehydrogenase, electron transfer subunit	11.74	52.16 ± 1.31
SSU05_1532	lipoprotein involved thiamine biosynthesis	2.49	5.76 ± 1.24
SSU05_1776	Permease	−7.31	−8.11 ± 1.16
SSU05_1815	Ribonucleases G and E	−7.40	−12.21 ± 1.01
SSU05_2154	Succinate dehydrogenase/fumarate reductase, flavoprotein subunit	−5.46	−3.14 ± 1.30

The transcription levels of 10 selected genes were compared between SC-19 and CΔ*stk* related to Δ*stk* (Figure S2B). The differential expression of these target genes of SC-19 and CΔ*stk* showed identical trend (five up-regulated and five down-regulated), although the fold changes were displayed significant different between SC-19 and CΔ*stk* (*p* < 0.05). This indicates that the STK complementation works but not completely restored.

### Functional perturbation in Δ*stk* vs. wild type SC-19

**Figure 4:**
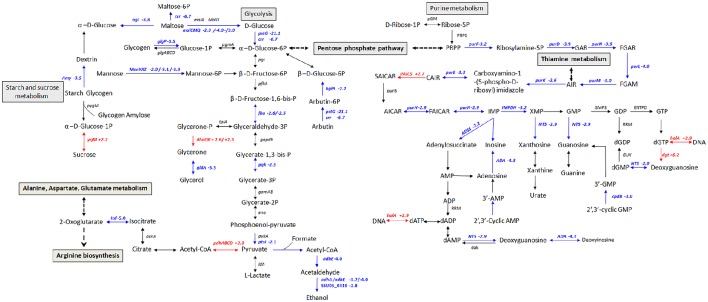
**Schematic representation of *S. suis* metabolic pathways differentially regulated in carbohydrate and purine metabolism**. Differentially expressed genes encoding proteins involved in glycolysis, starch, sucrose, and purine metabolism. Red color, up-regulated genes; blue color, down-regulated genes.

To get an overview of major perturbed functions in Δ*stk* compared with the wild type SC-19, a KEGG pathway enrichment analysis was conducted. Seven metabolic pathways were significantly repressed by *stk*-deletion (*q* < 0.1; Table 3). Among these, five pathways, namely, carbohydrate metabolism (ssu00010 and ssu00500), amino acid metabolism (ssu00290), nucleotide metabolism (ssu00230), PTS (ssu02060), as well as the other two pathways (ssu03010 and ssu00970) are crucial for the central metabolism of bacterial cells and are vital in translation. Most genes in the glycolysis were significantly down-regulated, including *ptsG* (SSU05_0397), *crr* (SSU05_0398), *pgk* (SSU05_0157), *adhA* (SSU05_0279), and *adhE* (SSU05_0280) (Figure 4). Notably, expression levels of *rpsN* (SSU05_1535), *rpsR* (SSU05_1832), *rpsP* (SSU05_0796), *rpsU* (SSU05_1433), and *glyS* (SSU05_1764) translation-associated genes in the pathways of ribosome and aminoacyl-tRNA biosynthesis were decreased in Δ*stk*. A total of 78% (28 out of 36) involved in various PTSs were down-regulated, including *manX/Y/Z* (SSU05_1778–1780)—encoding mannose transporter, *ptsG/crr/bglF* (SSU05_0397, SSU05_0398, and SSU05_1490) encoding maltose/glucose transporter, *celC* (SSU05_0709) encoding cellobiose transporter, and *ulaA/ulaB* (SSU05_2062, and SSU05_2063) encoding ascorbate transporter. Down-regulation of these pathways and related genes were potentially consistent with the decreased bacterial cell growth in the mutant strain.

**Table 3:** **Perturbated pathways significantly repressed in the Δ*stk* mutant strain**.

**Pathway**	***q*.val**	**Set.size**	**Pathway ID**
**CARBOHYDRATE METABOLISM**			
Glycolysis/Gluconeogenesis	0.086	27	ssu00010
Starch and sucrose metabolism	0.010	38	ssu00500
**TRANSLATION**			
Ribosome	0.002	53	ssu03010
Aminoacyl-tRNA biosynthesis	0.010	25	ssu00970
**MEMBRANE TRANSPORT**			
Phosphotransferase system (PTS)	0.010	35	ssu02060
**AMINO ACID METABOLISM**			
Valine, leucine, and isoleucine biosynthesis	0.063	11	ssu00290
**NUCLEOTIDE METABOLISM**			
Purine metabolism	0.086	54	ssu00230

### Virulence-associated genes

The *stk* deletion significantly reduced virulence of *S. suis*. Among 180 VAGs predicted by VFDB and known VAGs of *S. suis* (Fittipaldi et al., 2012), 52 genes were DEGs in Δ*stk*, including 32 down-regulated genes and 20 up-regulated genes (Table 4). These DEGs were mainly assigned into three classes: (i) 10 genes involved in adherence and immune evasion, including 9 down-regulated genes, such as *adsA* (SSU05_1000), *clpC* (SSU05_0389), and *clpE* (SSU05_0390); (ii) 17 genes involved in metal ion uptake, including 11 down-regulated genes, such as *hitC* (SSU05_1029), *fbpC* (SSU05_0551), and *mgtB* (ssu05_1418); (iii) 6 genes involved in ABC-type multidrug transport systems, including 5 up-regulated genes.

**Table 4:** **Virulence associated genes identified by RNA-Seq**.

**Gene name**	**Locus**	**Functions**	**Putative or confirmed function in virulence**	**Fold change**	**Up/Down**	***p*-value**
*hitC*	SSU05_1029	Iron ABC transporter, ATP-binding protein	HitABC	7.23	down	0
*hitC*	SSU05_1543	Iron ABC transporter, ATP-binding protein	HitABC	3.37	down	0
*hitC*	SSU05_0496	Iron ABC transporter, ATP-binding protein	HitABC	2.56	down	9.77E-77
*hitC*	SSU05_1882	Iron ABC transporter, ATP-binding protein	HitABC	2.66	down	8.68E-26
*fbpC*	SSU05_1253	Iron ABC transporter, ATP-binding protein	FbpABC	2.60	down	2.89E-77
*fbpC*	SSU05_0551	Iron ABC transporter, ATP-binding protein	FbpABC	3.54	down	0
*fbpC*	SSU05_2067	Iron ABC transporter, ATP-binding protein	FbpABC	2.28	down	0
*fbpC*	SSU05_1544	Iron ABC transporter, ATP-binding protein	FbpABC	2.16	down	4.17E-28
*feoB*	SSU05_1409	Ferrous iron transporter	FeoAB	2.79	down	0
*mgtB*	SSU05_1418	Mg2+ transport protein	MgtBC	2.65	down	0
*mgtB*	SSU05_1419	Mg2+ transport protein	MgtBC	2.12	down	1.08E-93
*pilB*	SSU05_2100	Major subunit PilB	PI-2a	3.42	down	1.5E-173
*pilB*	SSU05_2101	Major subunit PilB	PI-2a	3.78	down	1.91E-81
*sspA*	SSU05_0811	C5a peptidase precursor	C5a peptidase	2.43	down	9.31E-35
*sspA*	SSU05_0812	C5a peptidase precursor	C5a peptidase	2.53	down	2.23E-201
*permease*	SSU05_0798	Lipid transporter ATP-binding	LOS	2.08	down	2.52E-62
*cylG*	SSU05_1225	3-ketoacyl-ACP-reductase	Beta-hemolysin/cytolysin	2.01	down	0.000471076
*cwhA*	SSU05_0924	P60 extracellular protein, Invasion associated protein	p60	2.61	down	0.0114929
*adsA*	SSU05_1000	Adenosine synthase A	AdsA	2.85	down	0
*cyaB*	SSU05_0800	Cyclolysin secretion ATP-binding protein	Cya	3.37	down	2.68E-138
*bexA*	SSU05_0045	ATP-dependent polysaccharide export protein	Capsule	3.00	down	4.70E-08
*comGA*	SSU05_0126	General secretion pathway protein E	xcp secretion system	2.82	down	8.08E-05
*clpC*	SSU05_0389	Endopeptidase Clp ATP-binding chain C	ClpC	5.68	down	9.79E-56
*clpE*	SSU05_0390	ATP-dependent protease	ClpE	11.12	down	2.26E-95
*adhE*	SSU05_0280	acetaldehyde dehydrogenase	Lap	4.02	down	0
*purD*	SSU05_0033	Phosphoribosylamine-glycine ligase	Metabolism	3.87	down	0
*purA*	SSU05_1966	Adenylosuccinate synthase	Metabolism	2.15	down	0
*manN*	SSU05_1779	Mannose-specific PTS	Metabolism	3.89	down	0
*fhb*	SSU05_0272	H binding protein	Antiphagocytic	3.45	down	0
*ssnA*	SSU05_1968	DNA nuclease	Degradation of host DNA	3.53	down	0
*impdh*	SSU05_2183	Inosine 5′-monophosphate dehydrogenase	Metabolism	3.23	down	0
–	SSU05_0550	Glutamine ABC transporter	unknown	2.40	down	4.77E-178
*fbpC*	SSU05_0894	Iron ABC transporter, ATP-binding protein	FbpABC	4.40	up	8.28E-14
*fbpC*	SSU05_0891	Iron ABC transporter, ATP-binding protein	FbpABC	2.14	up	0
*fbpC*	SSU05_0946	Iron ABC transporter, ATP-binding protein	FbpABC	3.23	up	0
*hitC*	SSU05_0741	Iron ABC transporter, ATP-binding protein	HitABC	3.80	up	0
*CiaR*	SSU05_1095	Two component system response transcriptional positive regulator	PhoP	3.43	up	3.39E-12
*sboF*	SSU05_0894	Two component system response transcriptional positive regulator	PhoP	4.40	up	8.28E-14
*fepC*	SSU05_0650	Ferrienterobactin ABC transporter ATPase	Enterobactin	2.26	up	9.90E-13
*fepC*	SSU05_1669	Ferrienterobactin ABC transporter ATPase	Enterobactin	2.14	up	0
*metQ*	SSU05_1771	Immunogenic lipoprotein A	IlpA	2.80	up	0
*cps2J*	SSU05_0573	Glycosyl transferase	Capsule	2.44	up	1.07E-13
*sodB*	SSU05_1539	Superoxide dismutase	SodB	2.02	up	0
*gtrB*	SSU05_1004	Glycosyltransferase	LPS	2.78	up	2.54E-12
*permease*	SSU05_0947	Lipid transporter ATP-binding	LOS	5.33	up	2.44E-15
*permease*	SSU05_1405	Lipid transporter ATP-binding	LOS	3.97	up	0
*permease*	SSU05_1406	Lipid transporter ATP-binding	LOS	2.75	up	0
*permease*	SSU05_0911	Lipid transporter ATP-binding	LOS	2.61	up	0
*permease*	SSU05_0294	Lipid transporter ATP-binding	LOS	2.26	up	5.45E-13
*LuxS*	SSU05_0420	S-ribosyl homocysteinase	unknown	2.04	up	1.48E-11
*GtfA*	SSU05_1555	Glycosidase	unknown	2.94	up	0
–	SSU05_0053	Transcriptional regulator	unknown	2.17	up	0

### Analysis of phosphoproteomics

To investigate the Ser/Thr protein kinase activity and the substrates of STK in Δ*stk* and SC-19, we conducted phosphoproteomic analysis. In total, 32 phosphoproteins were detected. Of these 12 were differentially expressed phosphoproteins (DEPPs), including 9 down-regulated phosphoproteins (FC < 0.83) and 3 up-regulated phosphoproteins (FC > 1.2; Table 5; Table S4). As an internal control, auto-phosphorylation of STK was not detected in Δ*stk*. Among 12 DEPPs detected, all of the cell division associated proteins, including FtsA, GpsB, DivIVA, and MapZ were down-regulated. Another protein DnaK, which is a classical molecular chaperone, was down-regulated, as well as the predicted RNA-binding protein Jag. Moreover, down-regulated phosphorylation was detected in the predicted periplasmic solute-binding protein (SSU05_1717), an uncharacterized protein (SSU05_0066), and a hypothetical protein (SSU05_0636). Phosphorylation levels of three proteins, namely translation elongation factor (EF-Tu) involved in protein synthesis, fructose-bisphosphate aldolase (FBA) and GAPDH involved in glycolysis were up-regulated. Moreover, all of these three proteins were also involved in bacteria evasion of host defense, adhesion and invasion.

**Table 5:** **Differentially expressed phosphoproteins identified by phosphoproteomics**.

**Protein name**	**Locus**	**Uniprot ID**.	**Functions**	**Ratio (Δ*stk*/SC-19)**	**Phospho RS Site**	***p*-value**
FtsA	SSU05_0480	A0A075SC62	Actin-like ATPase involved in cell division	0.212	Ser310	0.043
GpsB	SSU05_0417	A4VZM2	Cell division initiation protein	0.215	Ser73	0.005
DivIVA	SSU05_0487	B9WXH0	Cell division initiation protein	0.209	Thr199	0.001
MapZ	SSU05_0419	B9WTU4	Mid-cell-anchored protein Z	0.057	Thr66	0.000
–	SSU05_1717	A0A075SSZ7	Predicted periplasmic solute-binding protein	0.151	Thr122/Thr197	0.005
Jag	SSU05_2013	B9WXK1	Predicted RNA-binding protein	0.054	Thr116/Thr158	0.045
DnaK	SSU05_0300	U5UDB7	Molecular chaperone	0.531	Thr591	0.029
EF-Tu	SSU05_0530	A4VZZ3	Translation elongation factor EF-Tu	1.442	Ser58	0.017
FBA	SSU05_0338	A0A0F7FJU8	Fructose-bisphosphate aldolase	1.235	Ser289	0.012
GAPDH	SSU05_0155	A2T9S8	Glyceraldehyde-3-phosphate dehydrogenase	1.696	Thr244	0.008
–	SSU05_0066	A4VYN7	Uncharacterized protein conserved in bacteria	0.037	Thr7	0.002
–	SSU05_0636	B9WVR3	hypothetical protein	0.089	Thr72	0.006

## Discussion

STK provides critical signaling that alters gene expression patterns in *S. pneumoniae* (Sasková et al., 2007), *S. mutans* (Banu et al., 2010), and *S. pyogenes* (Bugrysheva et al., 2011). In *S. suis*, STK is relevant in stress response and virulence. Down-regulating some VAGs in Δ*stk* mutant strain have been experimentally validated using qRT-PCR (Zhu et al., 2014). In this study, through “-omics” approaches, we found that *S. suis* STK can regulate expression of genes involved in bacterial central metabolism and virulence. Moreover, STK directly or indirectly affects phosphorylation of 12 proteins that are involved in cell division, glycolysis, and translation. These findings can explain the elongation of bacterial chain length, attenuated growth and virulence of *stk* deleted strain.

### Regulation by STK on cell growth and division

Inactivating *S*. *suis* STK affects the bacterial phenotypes, including cell chain length and growth kinetics (Figure 2). Consistently, the results have been reported in a previous study (Zhu et al., 2014). Several reports have also stated that *stk*-deficient could affect cell shapes and cell sizes in other Gram-positive bacteria, such as *S. pneumoniae* (Echenique et al., 2004; Fleurie et al., 2012), *S. aureus* (Donat et al., 2009), *S. mutans* (Banu et al., 2010), and *M. tuberculosis* (Kang et al., 2005).

In our study, we have used both OD_600_ value and CFU counts to measure the growth rate of SC-19, Δ*stk*, and CΔ*stk*. Through these two methods, we found during the mid-log phase, the growth rate of the three strains detected by OD_600_ value displayed no significant difference (Figure 2D), whereas the CFU counts showed the growth rate of Δ*stk* was much slower than SC-19 and CΔ*stk* during the mid-log phase (Figure 2E). Similar phenomenon has been also appeared in our previous study (Gao et al., 2016). The longer chains of the mutant strain may change the light-scattering properties of *S. suis* (Zheng et al., 2011) and other bacteria (Sha et al., 2004). Hence, growth detection through OD value was not adequate, and the CFU determination can accurately reflect the growth ability.

Phosphorylation of factors involved in cell division could function as an internal clock that regulates the sequence and timing of the cell cycle events (Grangeasse and Lesterlin, 2015). According to the phosphoproteomic analysis in this study, phosphoproteins associated with cell growth and division, such as DivIVA and MapZ, were down-regulated in Δ*stk* (Table 5). This provides an explanation for the weakened growth ability and elongated chain length of Δ*stk*. DivIVA has been identified as a STK-substrate in *S. pneumoniae* (Nováková et al., 2010). Moreover, *S. pneumoniae* with the non-phosphoablative form of DivIVA possess an elongated shape with a polar bulge and aberrant spatial organization of nascent peptidoglycan (Fleurie et al., 2012). These findings suggest that phosphoablative form of DivIVA is crucial for maintaining cell shape and division. MapZ is a newly discovered cell division protein essential in proper septum placement and likely functions as a marker of the cell division site (Holecková et al., 2014). *S. pneumoniae* with a non-phosphoablative form of MapZ exhibited cell shape and viability defects (Fleurie et al., 2014). Similar functions of DivIVA and MapZ may exist in *S. suis*. Hence, STK of *S. suis* may influence the cell growth and division of *S. suis* by regulating phosphorylation of DivIVA and MapZ. The other two down-regulated phosphoproteins in Δ*stk*, FtsA, and GpsB involved in cell division, may also contribute to the STK regulated cell division process. In *B. subtilis*, GpsB is phosphorylated by STK and its phosphorylation regulates STK activity through a negative feedback loop (Pompeo et al., 2015). In *E. coli*, FtsA is crucial in recruiting of FtsZ filaments to the membrane and negative regulated FtsZ organization (Loose and Mitchison, 2014).

### Regulation by STK on virulence

The deletion of *stk* in *S. suis* also resulted in the alteration of bacterial pathogenecity. This attenuation may result from the impaired growth of Δ*stk*, and also can be due to direct effect on the expression of virulence genes. As previously reported, *stk*-deletion strain displayed reduced ability to adhere to and invade in epithelial cells and increased sensitivity to phagocytosis (Zhu et al., 2014). In this study, analysis of gene expression profiles indicated that several genes associated with adherence and immune evasion were repressed in Δ*stk* (Table 4), such as *adsA* (−2.85-fold), *clpC* (−5.68-fold) and *clpE* (−11.12-fold). The gene *adsA* encodes an adenosine synthase AdsA. The *adsA* mutant of *B. anthracis* was easier to be cleared compared with the wild-type strain, implicating its role in immune evasion in the host (Thammavongsa et al., 2009). The gene *clpC* encodes a heat shock protein ClpC with ATPase activity. In *L. monocytogenes*, ClpC is a general stress response protein required for *in vivo* survival by promoting early bacterial escape from the phagosome of macrophages (Rouquette et al., 1998). Moreover, ClpC is required for adhesion and invasion. A *clpC*-deficient mutant of *S. pneumoniae* displayed decreased expression of CbpA (a structural adhesion) and pneumolysin (Nair et al., 2000). *clpE* acts synergistically with *clpC* in virulence and the *clpE* mutant of *L. monocytogenes* exhibited a significant reduction in virulence (Nair et al., 1999). Therefore, our results indicated that these down-regulated VAGs potentially contributed to decreased adherence to epithelial cells, increased immune evasion and increased sensitivity to phagocytosis of Δ*stk*.

The Δ*stk* mutants displayed defects in their ability to adapt to various environmental conditions, such as high temperature, high osmotic, oxidative, and low acidic pH stress (Zhu et al., 2014). These may due to the down-regulation of 11 genes encoding iron ABC transporters (HitC/FbpC, MgtB) that mediate the uptake of nutrients and several metabolism-related enzymes (i.e., ManN, PurA, and IMPDH; Table 4). *S. suis* requires nutrients, including trace metals, whose availability within the infected host is relatively low. The *hitC/fbpC* operon encodes an iron transport system that is responsible for utilizing iron bound to transferrin or iron chelates (Sanders et al., 1994). Mg^2+^ transporters encoded by *mgtB* are unique transport systems with unusual mechanisms for mediating Mg^2+^ movement through the membrane (Moncrief and Maguire, 1999). *manN* (−3.89-fold), whose product is the component of the mannose-specific PTS, can regulate hemolysin gene expression (Wilson et al., 2007). Down-regulation of these VAGs may hinder the acquisition of nutrients of *S. suis*, hence decrease the adaptation of *S*. *suis* to various conditions.

Other VAGs of *S*. *suis*, such as *fhb* (−3.45-fold) and *ssnA* (−3.53-fold), have been identified previously. The gene *fhb* encodes a factor H binding protein, which is a major antiphagocytic factor in preventing C3b deposition and blocking activation of the alternative pathway of the complement system (Pian et al., 2012). The cell wall-anchored DNase encoded by *ssnA* plays a role in disruption of neutrophil extracellular traps (De Buhr et al., 2014). Down-regulation of *fhb* and *ssnA* may be associated with the attenuated virulence.

According to the phosphoproteomic analysis, the phosphorylation levels of three proteins associated with virulence were up-regulated in the Δ*stk* (Table 4). They are EF-Tu, GAPDH, and FBA. Interestingly, all of them are the bacterial outer surface proteins that contribute to evasion of host defense, adhesion and invasion (Tan et al., 2008; Li Q. et al., 2015). Ser/Thr phosphorylation of EF-Tu has been reported in *E. coli, Thermus thermophilus, Streptomyces coelicolor*, and *B*. *subtilis* (Pereira et al., 2011). EF-Tu phosphorylation could accelerate its release from the translation site (Absalon et al., 2009). In *B. subtilis*, phosphorylation impairs the essential GTPase activity of EF-Tu by preventing its release from the ribosome, leading to the overall protein synthesis inhibition (Pereira et al., 2015). However, the role of EF-Tu phosphorylation in pathogen virulence has not been reported. GAPDH is the surface expression protein with the ability to bind albumin (Brassard et al., 2004), which serves as a receptor for the plasmin receptor and contributes to host cytoskeletal protein binding and signal transduction between bacteria and host tissues (Gase et al., 1996; Jobin et al., 2004). FBA catalyzes the reversible reaction of fructose 1,6-bisphosphate transferring into glyceraldehydes-3-phosphate and dihydroxyacetone (Jado et al., 1999). The effects of GAPDH and FBA phosphorylation on virulence remain unknown. The biological significance of phosphorylation modulation by STK on these virulence associated proteins deserves further investigations.

### Regulation by STK on metabolism

In our study, evaluating of the transcriptional status of gene pathways revealed that seven pathways are significantly repressed in Δ*stk* (Table 3). These repressions could be an important reason of the impaired growth of Δ*stk*.

Glycolysis is the most critical phase in glucose metabolism to produce energy in the form of ATP during bacterial cellular respiration (Kresge et al., 2005; Mulukutla et al., 2014). Our study found more than half of the genes (17 out of 27) involved in glycolysis were repressed in Δ*stk*, such as *crr* (−6.67-fold), *bglA* (−4.61-fold), and *pstG* (−20.63-fold). The gene *crr*, which encodes a glucose-specific phosphotransferase enzyme component for glucose transport into the cytoplasm, is involved in glycolysis, starch and sucrose metabolism (Han et al., 2007). *bglA* encodes a 6-phospho-β-glucosidase to convert host derived-sugars into usable monosaccharaides that contribute to energy generation for bacterial survival (Terra et al., 2015). *pstG* codes for a glucose transporter that enables catalysis of glucose to transform into glucose-6p (Buhr et al., 1994). Down-regulating the enzymes mentioned above in Δ*stk* may hinder glucose utilization, nutrient storage, and ATP synthesis.

More than two thirds of the genes (34 out of 54) involved in the purine metabolism pathway were repressed in Δ*stk* (Table S3). Previous studies have emphasized the importance of nucleotide biosynthesis in bacteria. Auxotroph mutants of nucleotide biosynthesis in *Salmonella* (McFarland and Stocker, 1987), *S. aureus* (Mei et al., 1997), and *S. pneumoniae* (Polissi et al., 1998) have shown impaired growth.

Our results showed that translation-associated pathways (ssu00970 and ssu03010) are the most affected pathways in Δ*stk*, which has not been reported in other bacteria (Table 3). In Δ*stk*, 88% of the genes involved in ribosomes (ssu03010) were decreased (46 out of 52), and in aminoacyl-tRNA biosynthesis (ssu00970), 84% of the genes were decreased (21 out of 25). The ribosome plays a major role in determining the overall gene expression profile of the cell (Starosta et al., 2014). Meanwhile, *glyS* (−2.51-fold) encoding a glycyl-tRNA synthetase has been suggested to be involved in DNA stabilization under extreme environmental conditions (Kumar et al., 2011). It has been previously shown that alterations of genes involved in translation can provoke slower growth of *E*. *coli* (Ruusala et al., 1984; Starosta et al., 2014).

Phosphoproteome analysis in this study has revealed that the phosphorylations of two glycolytic enzymes (FBA, GAPDH) and a translation asscociated protein EF-Tu were up-regulated (Table 4). These proteins are also involved in *S. suis* virulence, which have been discussed above. The phosphorylation of a chaperone DnaK was down-regulated in Δ*stk*. DnaK is induced in *E*. *coli* upon heat shock and promotes ATP-dependent refolding or degradation of damaged proteins (Sherman and Goldberg, 1993). Previously, *in vivo* phosphorylation has been shown to enhance DnaK binding to polypeptide substrates (Panagiotidis et al., 1994). Modulations of phosphorylation levels on these proteins may also lead to impaired metabolism of Δ*stk*.

## Conclusion

In our study, genome-wide transcriptional analyses identified 32 down-regulated VAGs in Δ*stk* compared with the wild type strain. Additionally, seven pathways were significantly repressed in Δ*stk*, particularly the translation-associated pathways. Phosphoproteomic analysis found that post-translational modifications of 12 proteins were significantly affected in Δ*stk*. These DEPPs are involved in various biological processes, including cell growth and division, glycolysis, and translation. Consistently, phenotypic assays confirmed that the Δ*stk* strain displayed deficient growth and attenuated pathogenicity. Therefore, STK-mediated signal transduction is important in cell growth and division, and metabolism of *S. suis*.

## Author contributions

RZ conceived and designed this project and experiments. CZ, WS, MT, MD, and WL performed the experiments. CZ, TG, LL analyzed the data and contributed to the development of the figures and tables. CZ, RZ, and ZX wrote the manuscript. All authors reviewed the manuscript.

### Conflict of interest statement

The authors declare that the research was conducted in the absence of any commercial or financial relationships that could be construed as a potential conflict of interest.
