# Supplementary material for: The Eukaryote-Like Serine/Threonine Kinase STK Regulates the Growth and Metabolism of Zoonotic Streptococcus suis
Source: Front Cell Infect Microbiol. 2017 Mar 7;7:66. doi: 10.3389/fcimb.2017.00066 (PMC5339665; doi:10.3389/fcimb.2017.00066)
Supplement: Supplementary file 1 [file Table1.DOCX]

**TABLE S1│ Oligonucleotide primers used in this study**

| **Primers** | **Primers sequence(5’-3’)*** | **Functions or PCR product** |
| --- | --- | --- |
| SU-F | AGAAGCTTGAAAATTGGTGGTATAATAACCT | Upstream border of *stk* |
| SU-R | GCGTCGACTACCTAGCCTCCTCCGTAAT |  |
| SD-F | AGGGATCCAGATGAATAAGGTTGGGGAG | Downstream border of *stk* |
| SD-R | CGGAGCTCAATATGATTCACCCAGGAAAC |  |
| Erm-F | AGGTCGACGAGTGTGTTGATAG | Erm^R^ expression cassette |
| Erm-R | GTGGATCCCTTGGAAGCTGTCAG |  |
| CS-F | GCGCGGATCCATGATTCAAATCGGTAAGATCTT | ORF of *stk* gene |
| CS-R | GGTGAATTCTTATTGTCCGCTACCTGTTG |  |
| IM-F | ACATGCATGCATGGAGGCAGGACAGGTAT | Exogenous promoter of *stk* |
| IM-R | GCGCGGATCCGTTCTTTCTTTTGGG |  |
| *stk*-F | CGGAATTCGCATGATTCAAATCGGTAAGATC | ORF of *stk* gene |
| *stk*-R | CGCTCGAGTTATTGTCCGCTACCTGTTG |  |
| SSU05_0427-F | ATGGCTTTTTCATCGGTCAACTTAG | Upstream gene of *stk* |
| SSU05_0427-R | TTACCTAGCCTCCTCCGTAATGTGA |  |
| SSU05_0429-F | TTGGATGGAGTTCTTTTACTTATTT | Downstream gene of *stk* |
| SSU05_0429-R | TCATACGCGAACCACCTCCACATCT |  |
| 16SrRNA-F | GTAGTCCACGCCGTAAACG | For real-time PCR |
| 16SrRNA-R | TAAACCACATGCTCCACCGC |  |
| SSU05_1776-F | CTTCTTTACCTATACAGTGG |  |
| SSU05_1776-R | CTTGATAATACTTACGAAC |  |
| SSU05_0272-F | GAGGTTACTGAGCCATCTT |  |
| SSU05_0272-R | TCCGTATTATCTGAAGGTTC |  |
| SSU05_1815-F | ACTACTCCTCGGACCAAC |  |
| SSU05_1815-R | AACAAGGATATGTGGAAAGTC |  |
| SSU05_0309-F | CTTAGACACCGCAGCCAT |  |
| SSU05_0309-R | GGATTCGTCAAAGATGGATT |  |
| SSU05_0792-F | CTTGGTGGTCAGACTGG |  |
| SSU05_0792-R | ACAGTTTAGTTCCCAACAAC |  |
| SSU05_0358-F | AGAAATGGATGTGTTTGTGAG |  |
| SSU05_0358-R | GTGAAGTAGTTACTCGCC |  |
| SSU05_2154-F | GTAATACGGCTAAATCATCAG |  |
| SSU05_2154-R | CAATTCAGGATCGTTTGTCC |  |
| SSU05_1011-F | TATCCATATTCGTGTCCCAG |  |
| SSU05_1011-R | CCACTCGGTAAATAATCCG |  |
| SSU05_0906-F | GAAACGAGTAAAGCAAGAAAT |  |
| SSU05_0906-R | TGCATTAGTTCAATATGTTTGT |  |
| SSU05_1532-F | AACACAGTCTGGCTGAGG |  |
| SSU05_1532-R | CATTTCTTGGGTTGGAAGTT |  |
| *Underlined nucleotides denote enzyme restriction sites | | |
