# Supplementary material for: The Eukaryote-Like Serine/Threonine Kinase STK Regulates the Growth and Metabolism of Zoonotic Streptococcus suis
Source: Front Cell Infect Microbiol. 2017 Mar 7;7:66. doi: 10.3389/fcimb.2017.00066 (PMC5339665; doi:10.3389/fcimb.2017.00066)
Supplement: Supplementary file 5 [file Image1.pdf]

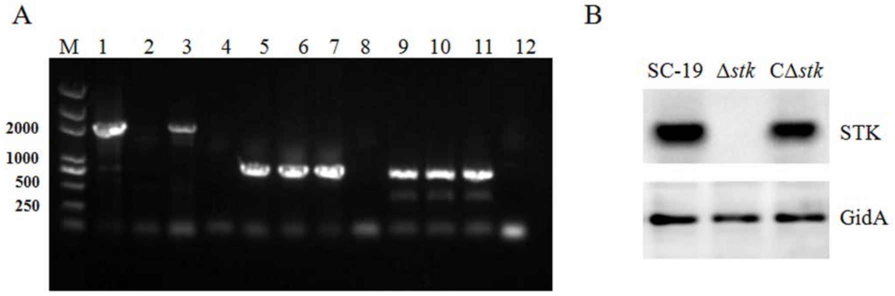

Figure S1 Confirmation of the isogenic mutant Δ*stk* and the complentary strain CΔ*stk*. (A) Confirmation of the Δ*stk* and CΔ*stk* by RT-PCR. Lanes 1-4 represent the amplification of *stk* using the primer set *stk*-F and *stk*-R. Lanes 5-8 represent the amplification of downstream gene of *stk* using the primer set 0427-F and 0427-R. Lanes 9-12 represent the amplification of upstream gene of *stk* using the primer set 0429-F and 0429-R. Lanes 1, 5 and 9 use cDNA of SC-19 as templates, lanes 2, 6 and 10 use cDNA of Δ*stk*, whereas lanes 3, 7, 11 use cDNA of CΔ*stk*. Lanes 4, 8, 12 represent the negative control. **(B) Confirmation of the Δ*stk* and CΔ*stk* by Western blot analysis.** The supernatant of cell lysate from SC-19, Δ*stk* and CΔ*stk* were disposed for immunoblot analysis with STK or GidA polyclonal antibodies. An antibody directed against GidA was used as loading control.
