# Supplementary material for: The Eukaryote-Like Serine/Threonine Kinase STK Regulates the Growth and Metabolism of Zoonotic Streptococcus suis
Source: Front Cell Infect Microbiol. 2017 Mar 7;7:66. doi: 10.3389/fcimb.2017.00066 (PMC5339665; doi:10.3389/fcimb.2017.00066)
Supplement: Supplementary file 6 [file Image2.pdf]

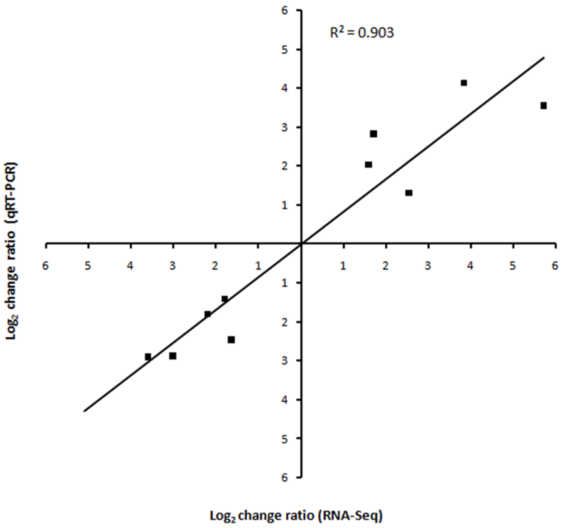

Figure S2A Correlation between the transcriptome data and the qRT-PCR results. The fold changes in transcript levels of 10 selected genes determined by both methods were $\log_2$ transformed, and the values were plotted against each other to evaluate their correlation.

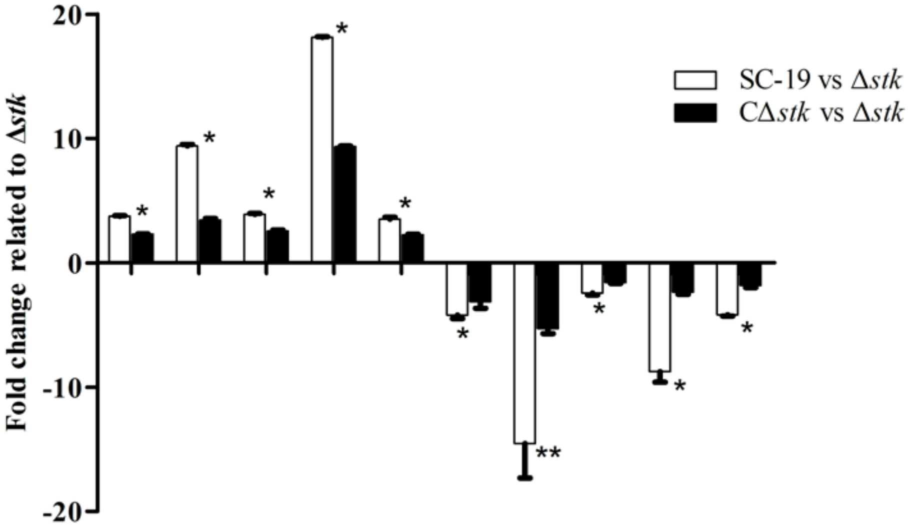

Figure S2B Comparision of transcription levels of selected genes of SC-19 and CΔ*stk* related to Δ*stk*. The fold changes in transcription levels of 10 selected genes determined by $\log_2$ transformed. Five depressed genes in Δ*stk* (from left to right: SSU05_1776, SSU05_1815, SSU05_2154, SSU05_0272, and SSU05_0309) and five induced genes in Δ*stk* (from left to right: SSU05_0358, SSU05_0792, SSU05_0906, SSU05_1011, and SSU05_1532). Statistical significance was determined using the two-tailed *t*-test (*$p < 0.05$; **$p < 0.01$).
